# Bottom-Up Copper Filling of Large Scale Through Silicon Vias for MEMS Technology

**Published:** 2018

**Authors:** L.A. Menk, D. Josell, T. P. Moffat, E. Baca, M. G. Blain, A. Smith, J. Dominguez, J. McClain, P. D. Yeh, A. E. Hollowell

**Affiliations:** 1 Microsystems and Engineering Sciences Applications (MESA) Complex, Sandia National Laboratories, Albuquerque, New Mexico 87123, USA; 2 Materials Science and Engineering Division, National Institute of Standards and Technology, Gaithersburg, Maryland 20899, USA

## Abstract

An electrodeposition process for void-free bottom-up filling of sub-millimeter scale through silicon vias (TSVs) with Cu is detailed. The 600 μm deep and nominally 125 μm diameter metallized vias were filled with Cu in less than 7 hours under potentiostatic control. The electrolyte is comprised of 1.25 mol/L CuSO_4_ −0.25 mol/L CH_3_SO_3_H with polyether and halide additions that selectively suppress metal deposition on the free surface and side walls. A brief qualitative discussion of the procedures used to identify and optimize the bottom-up void-free feature filling is presented.

Emerging 3-D MEMS devices span multiple length scales, and their fabrication often requires a combination of existing technology coupled with new processing developments and optimization thereof. Two widely practiced electrochemical metallization schemes of interest are through-mask deposition^1^ and Damascene processing.^2^ For the former, electrical contact is made to a common backplane and the growth front propagates in a bottom-up fashion guided by the insulating side walls of the template. An important limitation of this scheme is that, absent subsequent subtractive processing of the common backplane, the as-built structures are electrically tied. This feature makes fabrication of complex multilevel structures difficult if not impossible. In contrast, in Damascene processing the entire exposed template surface is first metallized and the recessed surface features are then filled by either superconformal^3^ or bottom-up^4,5^ electrodeposition processes whose growth dynamics derive from specific surfactant additives. The surface is planarized following feature filling to achieve the desired electrical isolation of the fabricated interconnects. The entire process may be repeated as needed to produce intricate multilevel structures of arbitrary connectivity. For different reentrant shapes and length scales the processing conditions need to be tuned and optimized to ensure that the additives inhibit deposition on the side walls and free surfaces while sustained active metal deposition proceeds on the bottom surface.^5–7^

In this work, the length scale of recessed features of interest falls between those of printed circuit board technology and TSV chip stacking. The via last process involved back side etching of vias across the 600 μm thick wafer to land on the conductive features on a silicon-on-insulator device layer as indicated in Figure 1. The thickness of the handle wafer was necessary to meet the stiffness requirements of the desired MEMS architectures and applications. Likewise, the width of the features is limited due to reactive ion etch (RIE) lag deep in the Si; the 125 μm diameter was selected to keep the aspect ratio low enough to enable reliable fabrication of the template and derived structures. To ensure all vias were viable required over-etching that is responsible for the large notches at the bottom corner of the TSVs. These create additional difficulties not only for depositing insulating liners and the conductive seed metal but also for subsequent electroplating.

The present challenge is to completely fill the 125 μm wide and 600 μm deep features (aspect ratio close to 5), lined with atomic layer deposited Pt, with electrodeposited Cu. To fill the features, a two component, polyether-chloride additive electroplating chemistry is adopted that was previously used for extreme bottom-up filling of smaller annular TSVs with 8 μm inner diameter, 19 μm outer diameter, and 56 μm depth.^4,7^

Non-linear bottom-up filling is a critical process that utilizes positive feedback between localized breakdown of the co-adsorbed polyether-Cl^−^ suppressor layer and its sustained disruption by metal deposition.^4–7^ A marker for such behavior is hysteretic voltammetry with the relevant potential regime defined by a negative differential resistance (NDR).^4,7^ The inhibitor breakdown response is classified as an S-shaped NDR based on its shape or trajectory in the potential-current domain. The link between NDR and pattern forming and/or oscillating electrochemical reactions has been noted for a range of systems.^8–12^ For high current density processes such as metal deposition, dissolution or electrocatalytic reactions it is not uncommon for the critical nature of NDR systems to be obscured by other cell impedances, most usually the electrolyte resistance. Nevertheless, metal deposition reactions leave their mark and examination of a planar electrode following cyclic voltammetry often reveals evidence of the bifurcation of the surface into zones of active deposition and passive behavior.^4,13^ Such Turing patterns derive from the mismatch between slow diffusional processes associated with additive transport relative to the instantaneous global electrical response of the system.^4–6,11,12^ Selection of the active zones may have a stochastic element but in many instances can be related to electrode defects. Most importantly, topographic patterning tools such as those used to form TSVs provide the means to guide the otherwise spontaneous bifurcation into active and passive zones engineered for practical applications. When the dimensions of the patterned topography dominate the local roughness, the attenuated suppressor flux to recessed surfaces, relative to the free surface, favors bifurcation with the active site located in the remote recessed regions, thereby giving rise to bottom-up feature filling.

Key experimental parameters for a robust filling process are: the halide and polyether concentration required for bottom-up feature filling, wafer pretreatment to ensure complete electrolyte wetting of recessed surface features, and well defined hydrodynamics across the work piece.^4–7^ Formation of an effective suppressor layer involves co-adsorption of halide and polyether and is thereby sensitive to the bulk concentration of each.^4,7,14^ Wetting of micrometer to millimeter scale recessed surface features can be challenging and a modified scheme for pre-wetting with a water soluble low surface tension solvent is used. Finally, as both the Cu^2+^ and the suppressor components can be subject to mixed kinetic-mass transport control during feature filling, defined hydrodynamics are required to ensure uniform completion of feature filling at the pattern and workpiece scale.

The large size of the MEMS TSVs motivates modifications of the CuSO_4_ - H_2_SO_4_ - NaCl electrolyte previously used for bottom-up filling. Specifically, to minimize the deposition time, the Cu^2+^ concentration was increased from 1.0 mol/L to 1.25 mol/L CuSO_4_. Due to the limitation on Cu^2+^ solubility the supporting electrolyte was changed from 0.5 mol/L H_2_SO_4_ to 0.25 mol/L CH_3_SO_3_H with the added benefits of slightly increasing the contribution of electromigration to Cu^2+^ transport and lowering of the hazardous materials profile.^15^ This particular strategy has been previously applied to sub-micrometer Damascene trenches and TSV with features as large as 50 μm in diameter and an aspect ratio of 4 being successfully filled using Cu(CH_3_SO_3_)_2_ - CH_3_SO_3_H electrolyte.^16,17^ Finally, due to concerns with Na contamination of an adjacent CMOS processing environment^18–20^ the chloride source was changed from NaCl to KCl (one might equally well use HCl).

A combination of voltammetry and optical imaging of the planar electrode is used, in combination with prior insights on bottom-up Cu filling, to define the additive concentration, hydrodynamic conditions and potential window suitable for bottom-up TSV filling. The utility of such guidance is tested in a series of TSV filling experiments under potentiostatic conditions.

## Experimental

### Electrolyte

All experiments were performed at room temperature using the 1.25 mol/L CuSO_4_ −0.25 mol/L CH_3_SO_3_H electrolyte with 1 mmol/L KCl and a variable concentration of a poloxamine suppressor, Tetronic 701, Ethylenediamine tetrakis (propoxylate-block-ethoxylate) tetrol, M.W. 3,600 (Sigma Aldrich). The influence of poloxamine concentrations (1–50) μmol/L on the voltammetric behavior was surveyed. Stock solutions of the respective constituents were prepared with 18.2 MΩ · cm water with the 1.0 mmol/L poloxamine in water being refrigerated to avoid phase separation. The experimental electrolytes were formulated immediately before use with the addition of the poloxamine stock solution and KCl to the 1.25 mol/L CuSO_4_ −0.25 mol/L CH_3_SO_3_H.

### Voltammetry

Cu deposition was examined by slow scan voltammetry utilizing a polished Pt rotating disk electrode (RDE) with a nominal surface area of 0.196 cm^2^. The Pt RDE was used to capture the behavior that might be anticipated during deposition in the ALD Pt-seeded TSV. A scan rate of 2 mV/sec was used and the RDE was rotated at 400 rpm (800π rad/min) thereby giving definition to the hydrodynamics and effective boundary layer thickness. The RDE tip (Pine Instruments, Inc.) was used in the as-received factory polished condition. Any Cu remaining on the Pt RDE after each voltammetric cycle was removed by immersion in 10% HNO_3_ solution for several minutes followed by rinsing with water. The experiments were conducted in a 150 mL glass cell using a 4.7 cm^2^ platinum wire counter electrode and a saturated potassium sulfate/mercurous sulfate/mercury reference electrode.

### TSV deposition

All via filling experiments were conducted using approximately 2 cm by 1 cm cleaved samples having 100 vias per sample, patterned and etched at a pitch of 250 μm. The vias are 600 μm deep and nominally 125 μm wide with the exact geometry varying somewhat between specimens due to etch rate variability across the 150 mm diameter wafers. During via fill experiments the samples were rotated using the RDE apparatus and a modified specimen holder. The rectangular wafer fragments were mounted with the short edge connected to a central spindle with the vias opening upward. During operation the specimen was rotated at 400 rpm in a manner analogous to a helicopter blade.

Robust wetting of the high aspect ratio vias by the electrolyte proved to be challenging. Experimentation revealed that pre-wetting with low surface tension water soluble solvents, such as ethanol or isoproponal, to be an effective solution to this problem. Specimens mounted on the spindle were immersed in a solvent filled beaker and transferred to a vacuum chamber for rough pumping. The solvent was pre-cooled to −18°C to minimize the vapor pressure. The sample was submerged for approximately 10 minutes until bubbles were no longer observed indicating full wetting. Subsequently, the sample was quickly transferred for immersion into the electroplating bath before significant evaporation occurred. Once connected to the rotator, the specimens were rotated for 1 to 2 minutes in the electrolyte at open circuit conditions to allow the solvent within the TSVs to be exchanged with the CuSO_4_ - CH_3_SO_3_H electrolyte before stepping the potential to the desired value for feature filling. Following Cu deposition, the via samples were encapsulated in epoxy and mechanically cross-sectioned and polished on pads using 60 μm down to 0.25 μm embedded diamond grit. The samples were imaged using an optical microscope to reveal the fill profile and the presence of any occluded voids. Sectioning aimed to capture the growth front and feature filling across the diameters of rows of vias, although some variability in centering was evident among the specimens.

## Results and Discussion

### Voltammetry

**Figure 2.**
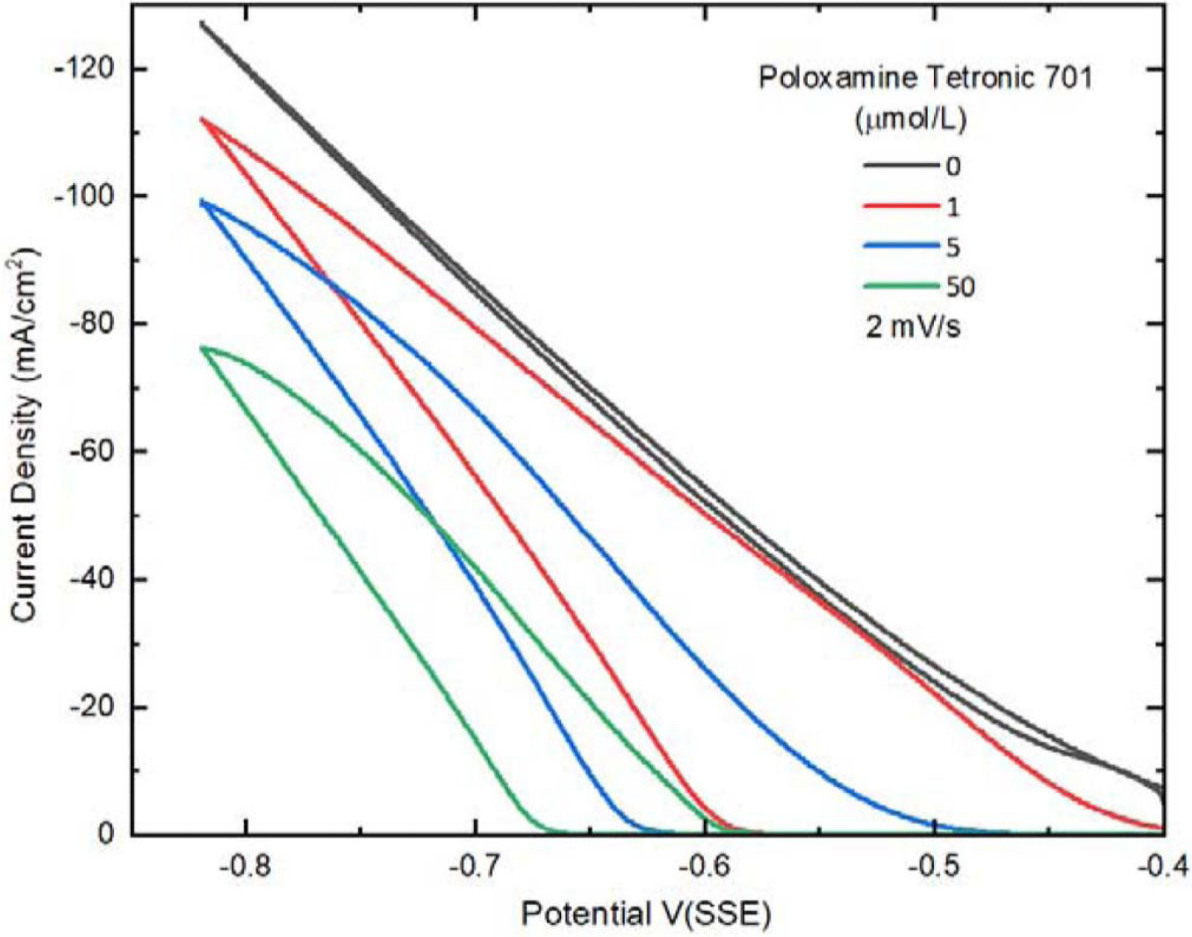
Cyclic voltammetry at 400 rpm showing the effect of adding poloxamine suppressor to the 1.25 mol/L CuSO_4_ −0.25 mol/L CH_3_SO_3_H-1 mmol/L KCl electrolyte. Without any suppressor, little hysteresis is evident between the reductive and oxidative directions of the scan, and significant Cu reduction occurs at a far less negative potential compared with solutions containing poloxamine Tetronic 701. As the poloxamine is added to the electrolyte, the onset of significant deposition that indicates suppressor breakdown shifts more negative, and increasing hysteresis is observed. The critical behavior of the suppression breakdown that underlies the S-NDR mechanism is hidden by the linear current-potential response associated with the impedance of the cell.

In 1.25 mol/L CuSO_4_ −0.25 mol/L CH_3_SO_3_H - 1 mmol/L KCl electrolyte, a smooth rise in current with polarization is evident in Figure 2. On the return scan the minimal hysteresis is consistent with little change of the surface chemistry during cycling as expected. At more negative potentials the response approaches linearity despite kinetics for Cu^2+^ reduction reaction that are known to follow an exponential dependence on overpotential. The difference reflects the significant contribution of electrolyte resistance to the overall voltammetric response. Evaluating the local slope at the negative potential limit yields a value of approximately ≈ 3 ohms · cm^2^ that, multiplied by the approximate current density of ≈ 0.1 A/cm^2^ yields a substantial ≈ 300 mV deviation between the applied potential and the overpotential that drives the metal deposition reaction.

The addition of 1 μmol/L Tetronic 701 results in significant inhibition of the deposition reaction evident as the shift in the onset of metal deposition of ≈ −0.18 V. Following the breakdown of suppression a rapid rise in the deposition rate with polarization is evident. The activation continues on the return sweep such that the response almost merges with the poloxamine-free curve, indicating near complete activation of the electrode surface. The hysteretic loop reflects the positive feedback derived from interplay of the breakdown of the inhibiting suppressor layer and the metal deposition reaction itself. Repeating the experiment with a freshly etched Pt RDE and a higher Tetronic concentration of 5 μmol/L reveals increased inhibition with further displacement of the onset potentials toward more negative values. Hysteresis is again seen with cycling although the net current on the return sweep no longer reaches that of the fully activated surface and the system repassivates near −0.48 V on the return sweep. Increasing the Tetronic concentration by an order of magnitude to 50 μmol/L continues the above trend, with suppression breakdown occurring at the more negative −0.67 V followed by acceleration of the deposition rate and hysteresis before subsequent repassivation near −0.59 V. The steeper slope associated with suppressor breakdown, compared to the ohmic-limited value for the additive-free case, is congruent with the critical nature of the suppressor breakdown and S-NDR behavior that is obscured by the resistive contribution associated with current flow through the resistive electrolyte. The results are analogous to those previously reported for sulfate-based electrolytes.^4^

**Figure 3.**
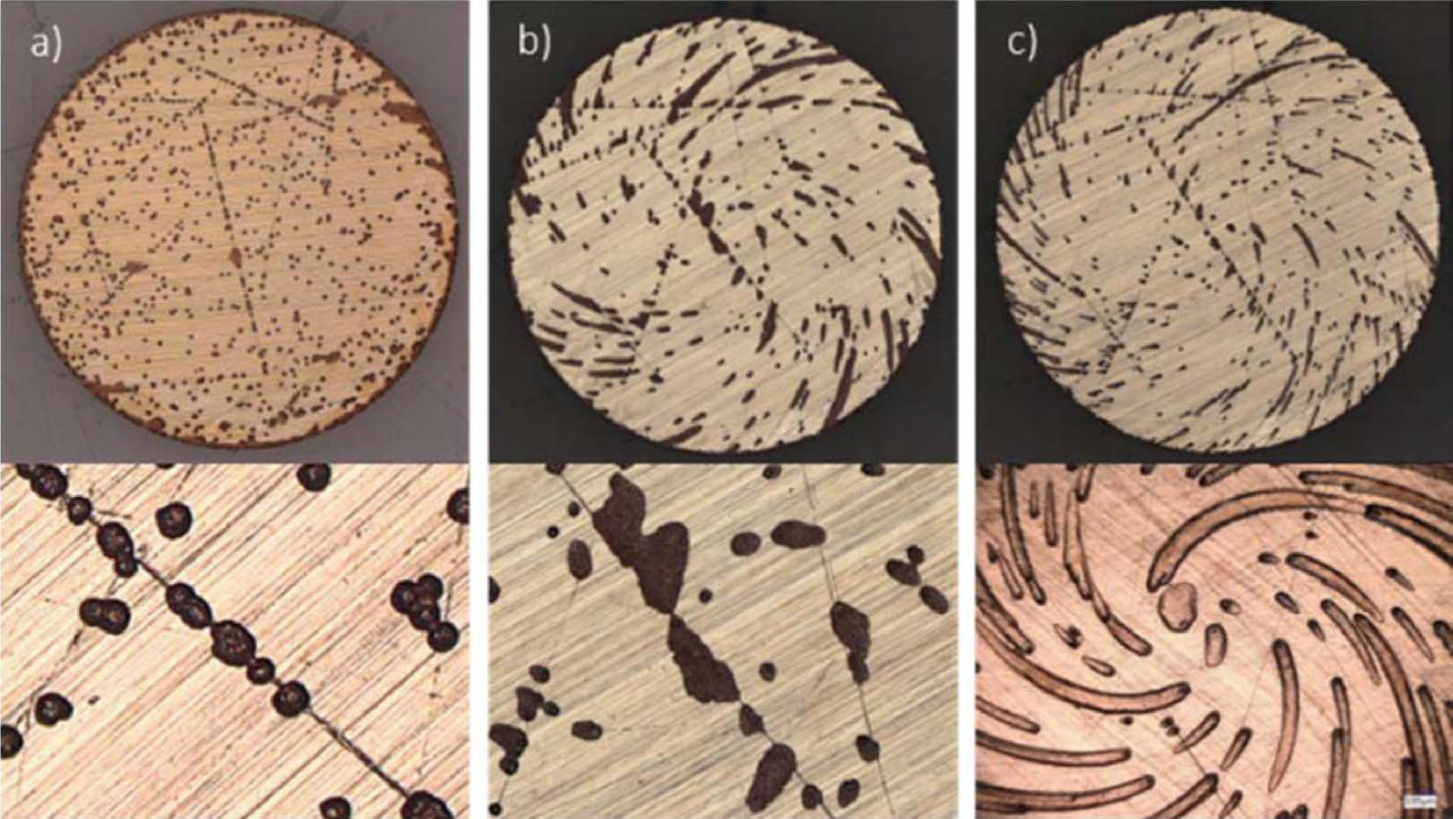
Deposition during cycle voltammetry on the 12 mm diameter RDE is non-uniform. Cycling between −0.4 V and −0.82 V (SSE) results in the development of Turing patterns as the electrode bifurcates into passive (bright) and active (dark) regions. The dark regions correspond to the growth of deposits that preferentially initiate at deeper abrasion on the electrode surface and then develop a hemicylindrical or hemispherical growth front. The Turing patterns are significantly affected by fluid flow as indicated in the respective images a.) 25 rpm, b.) 100 rpm and c.) 400 rpm. The high magnification image in (c.) is from a different experiment than the low magnification image, both conducted under the stated conditions.

Congruent with the above interpretation, optical examination of the RDE electrodes following one voltammetric cycle in the presence of 50 μmol/L Tetronic additive reveals bifurcation of the electrode into active and passive regions as shown in Figure 3. The influence of hydrodynamics was examined by repeating the experiment using the same Pt RDE, subject only to the chemical etching of residual Cu between runs, thereby allowing a more detailed comparison between the images regarding defects sites. At 25 rpm the active metal deposition zones are hemispherical in nature and distributed in two populations, one with locations that are uncorrelated and nominally random and the other clearly correlated with larger polishing scratches that correspond to the most recessed surface features. Repeating the experiment at 100 rpm further reveals the impact of the flow field on the shape and evolution of the active zones that are aligned with the laminar flow lines, including the subpopulation that nucleates preferentially on the major polishing scratches. Close inspection of the images in Figures 3a-3c for different hydrodynamic conditions reveals the plurality of nucleation sites for the active zones are associated with the same defects sites on the electrode, with the most definitive correlations apparent for the deepest scratches.

**Figure 4.**
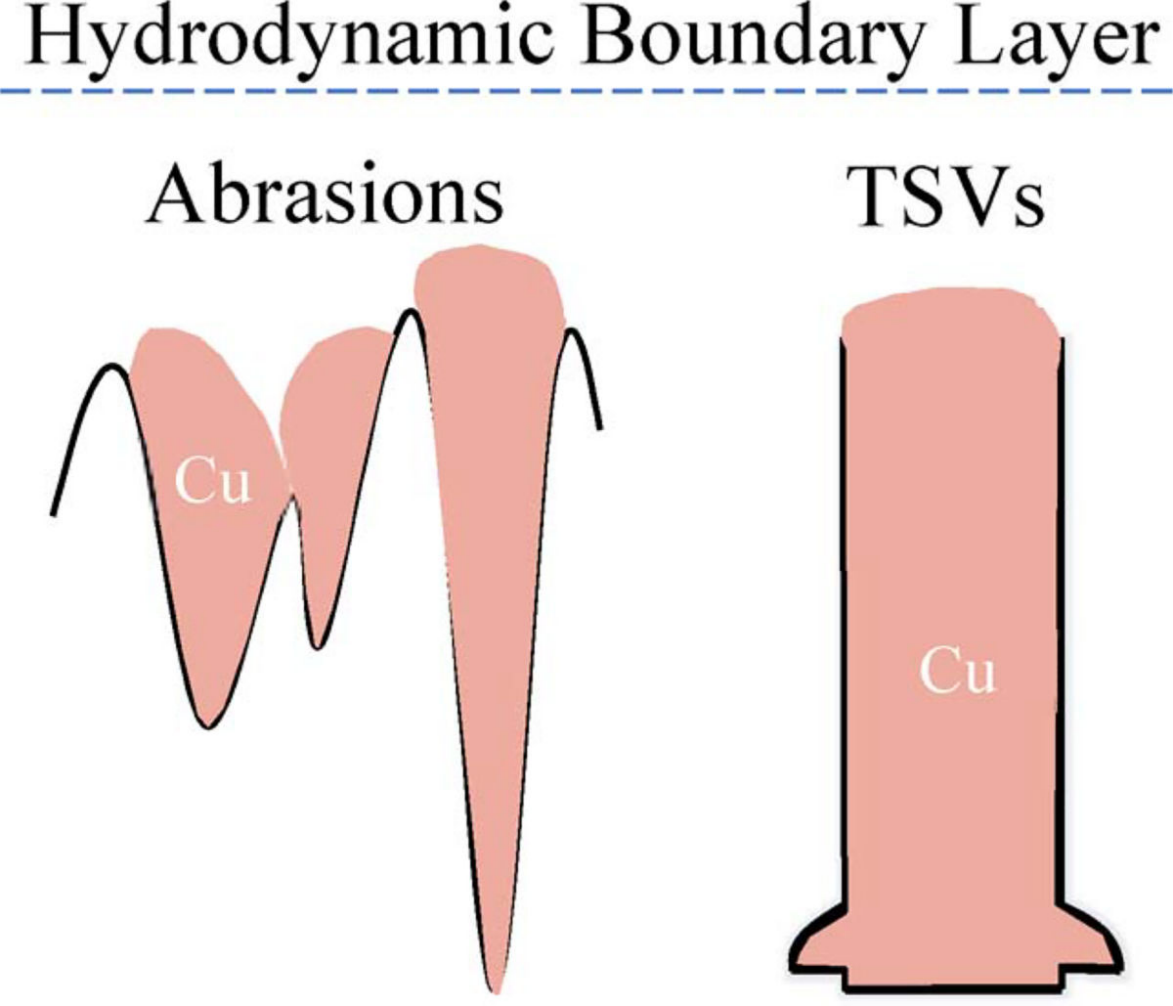
Sketch of the analogous Cu growth in different recessed features. The feature geometry and poloxamine Tetronic 701 concentration enable the bottom-up Cu filling, although convection may also affect deposition, as demonstrated in Fig. 3.

The selection of the most recessed surface features for active zone development is a direct analogy to the selective filling of engineered TSV. The deep features with high aspect ratios compared to the nonconforming comparatively flat boundary layer, shown schematically in Figure 4, results in a diminished flux of the suppressor species to the most recessed surface segments and initiation of bottom-up filling is anticipated. The process is history dependent and for regions where the metal deposition reactions occurs the ability of the suppressor film to reform on the dynamically moving surface segments is limited. This is further convolved with distribution of the applied potential between driving the metal deposition reaction versus ohmic losses in the electrolyte. Previous work with the NDR system indicates that for a given cell geometry and hydrodynamics bottom-up filling occurs when the workpiece potential is set at a value within the hysteretic voltammetric window, namely between the breakdown potential and its reformation value.^4-7^

**Figure 5.**
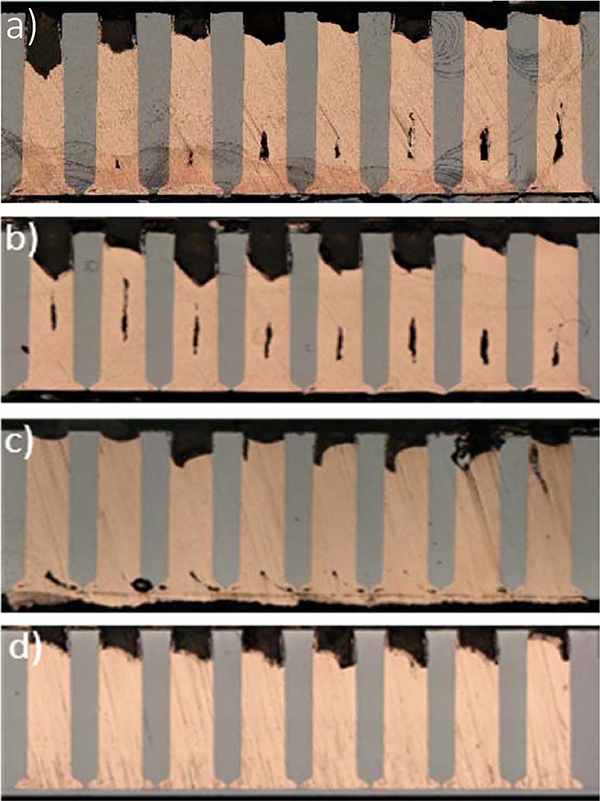
TSV filling after 6 hrs at a) −0.74 V(SSE), b) −0.70 V(SSE), c) −0.66 V(SSE), d) −0.64 V(SSE) in 1.25 mol/L CuSO_4_ −0.25 mol/L CH_3_SO_3_H − 1 mmol/L Cl^−^ electrolyte with 50 μmol/L poloxamine Tetronic 701. The vias were electroplated on approximately 1 cm by 2 cm samples and rotated at 400 rpm.

This criterion is tested by examining the filling of the large scale TSV at various potential in the presence of 50 μmol/L poloxamine. According to Figure 2 the potential window lies between suppressor breakdown at −0.67 V and the repassivation value at −0.59 V. Deposition at four different values of applied potential was examined. As shown in Figure 5 void free bottom-up filling is accomplished at −0.64 V and possibly −0.66 V. In the latter case, the voided material near the TSV bottoms may be a patterning artifact associated with the merging of the severely overetched TSV. In contrast, centerline voids are evident for deposition at −0.7 V and −0.74 V which lie beyond the specified voltammetric limits. Reduced voiding toward the left-hand side of the −0.74 V specimen might reflect the metallographic section not evenly capturing the full cross-section diameter of the vias or, alternatively, a patterning effect as suggested by the reduced fill height in these features. Thus, as with smaller vias, the voltammetrically determined breakdown and repassivation potentials serve as an effective tool for identifying the potentials for bottom-up deposition. It should be noted that the breakdown potential is primarily determined by the polyether-halide additive concentration while the repassivation process is very sensitive to hydrodynamics.^7^

**Figure 6.**
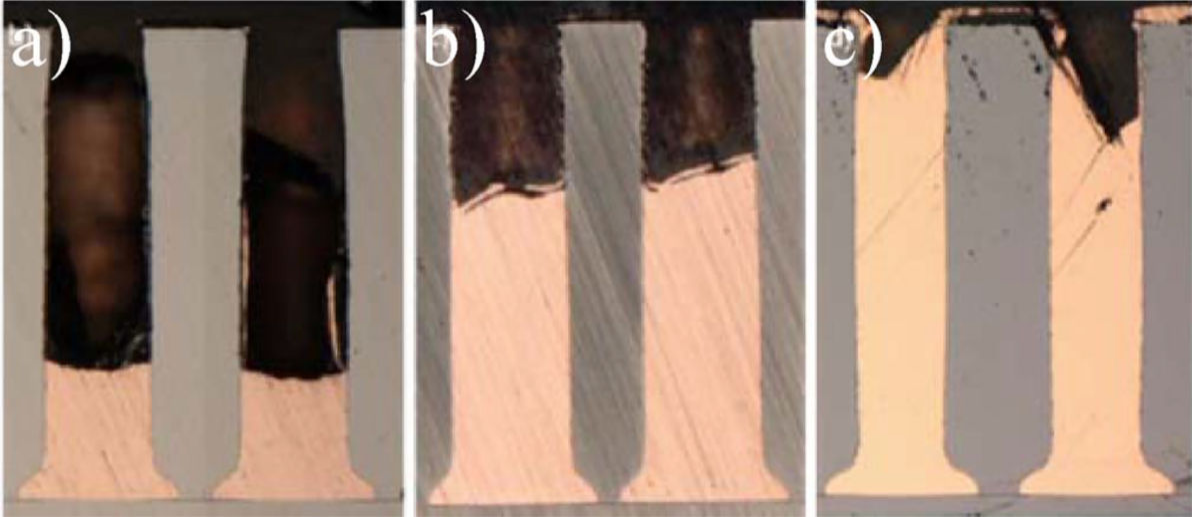
TSV filling at −0.64 V(SSE) for a) 2 hr, b) 4 hr, c) 6 hr in 1.25 mol/L CuSO_4_ −0.25 mol/L CH_3_SO_3_H − 1 mmol/L Cl^−^ electrolyte with 50 μmol/L poloxamine Tetronic 701. The vias were electroplated on approximately 1 cm by 2 cm samples and rotated at 400 rpm.

The most robust demonstration of bottom-up filling comes from examining its temporal evolution. This is shown in Figure 6 for a series of samples deposited at −0.64 V for times ranging from 2 hr to 6 hr. The bottom-up growth rate of nearly 100 μm/hr corresponds to a local current density approaching 75 mA/cm^2^. It is interesting that after 2 hours the growth front is flat and at a similar position within neighboring vias while at later times the growth begins to deviate from planarity and become asymmetric within each via. This indicates that the hydrodynamic flow field began to significantly impact via filling as the growth front approached its upper reaches.

To examine this question in more detail a computational study of the hydrodynamics was performed. Flow simulation were performed on a single cylindrical via in a cylindrical domain. The incompressible Navier-Stokes equations were solved numerically using ANSYS FLUENT, a finite volume method. The fluid flow equations were solved in a rotating reference frame with an angular velocity of 40 rad/s for the vias of interest which were located ≈ 2.5 cm from the axis of spindle rotation. The rotating frame was treated within FLUENT by the addition of appropriate body force terms in the fluid flow equations. The velocity boundary condition at the top of the via was 100 cm/s in the tangential (x) direction (consistent with the rotation rate), while the remaining surfaces were treated as walls with no-slip boundary conditions. The flow within the via behaved as a lid driven cylindrical cavity flow. The fluid viscosity and density were assumed to be that of water at room temperature.

**Figure 7.**
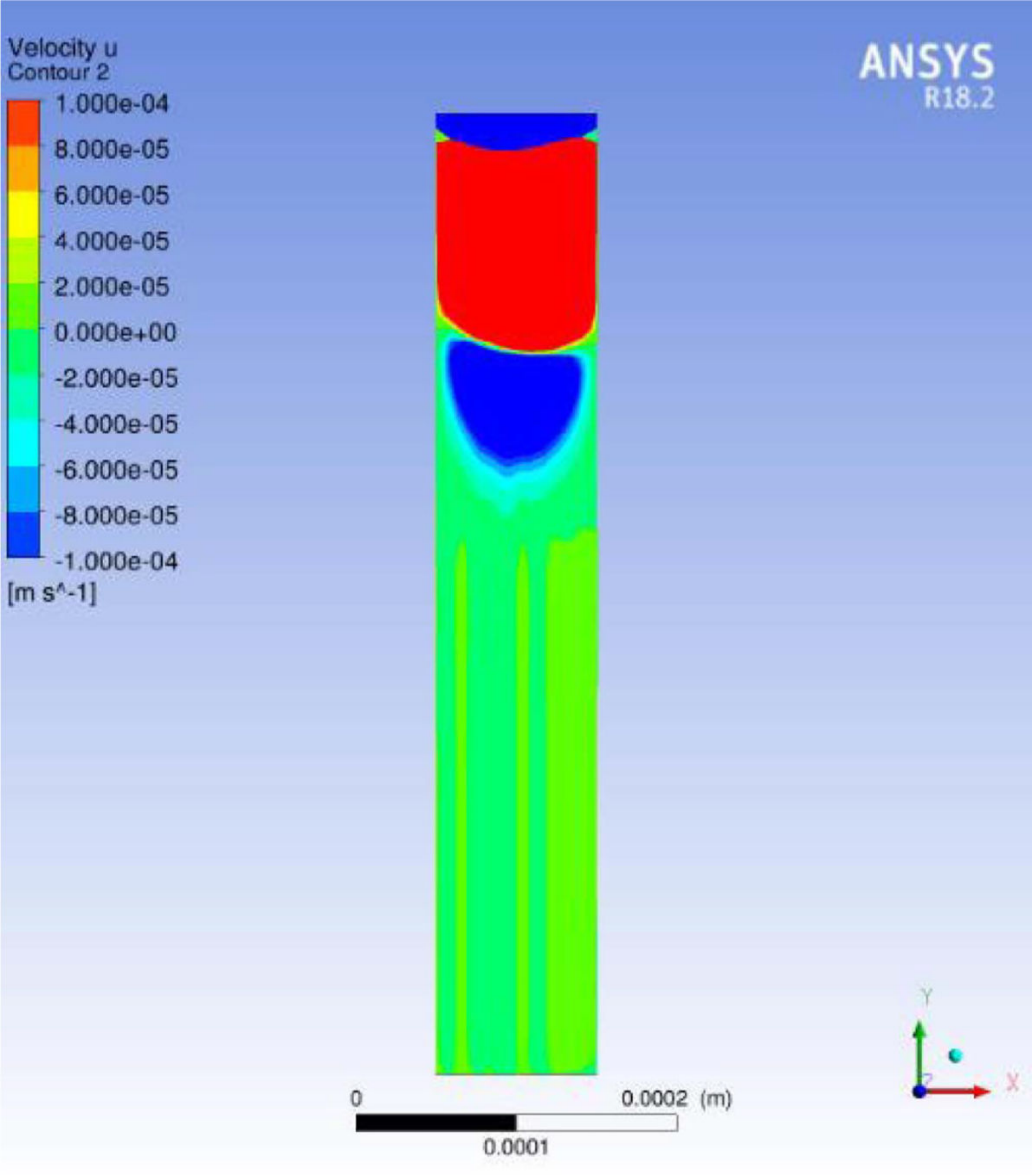
Flow simulation on a single cylindrical via using incompressible Navier-Stokes equations solved numerically with a finite volume method in ANSYS FLUENT on a cylindrical domain. The fluid flow equations are solved in a rotating reference frame with an angular velocity of 40 rad/s for an axis of rotation that is parallel to the via and displaced to its side by 2.5 cm to simulate the location of an experimental via on a rotating wafer piece.

Figure 7 shows the x-component of the velocity field within the xy-plane that cuts through the midplane of the via and is parallel to the local flow over the via. The alternating colors of x-velocity indicate the presence of stacked counter-rotating vortices expected with cavity flow at low Reynolds numbers. The flow structures observed in the fluid simulations appear to correlate with the asymmetric filling that is evident near the tops of the TSV in Figures 5 and 6, indicating that the deposition pattern may be influenced by the flow field near the top of the via. In contrast, the fluid is nearly stationary in the bottom two-thirds of the via. Thus, while solution replenishment due to fluid flow affects the fill profile in the upper third of the via, transport is dominated by diffusion farther down congruent with the experimentally observed growth front geometry.

The growth profiles in Figures 5 and 6 and the flow profile in Figure 7 indicate that further attention will be needed to address the impact of convective contributions, including both the formation of vortices and boundary layer dimensions, on the uniformity of TSV filling for a successful wafer plating process.

**Figure 8.**
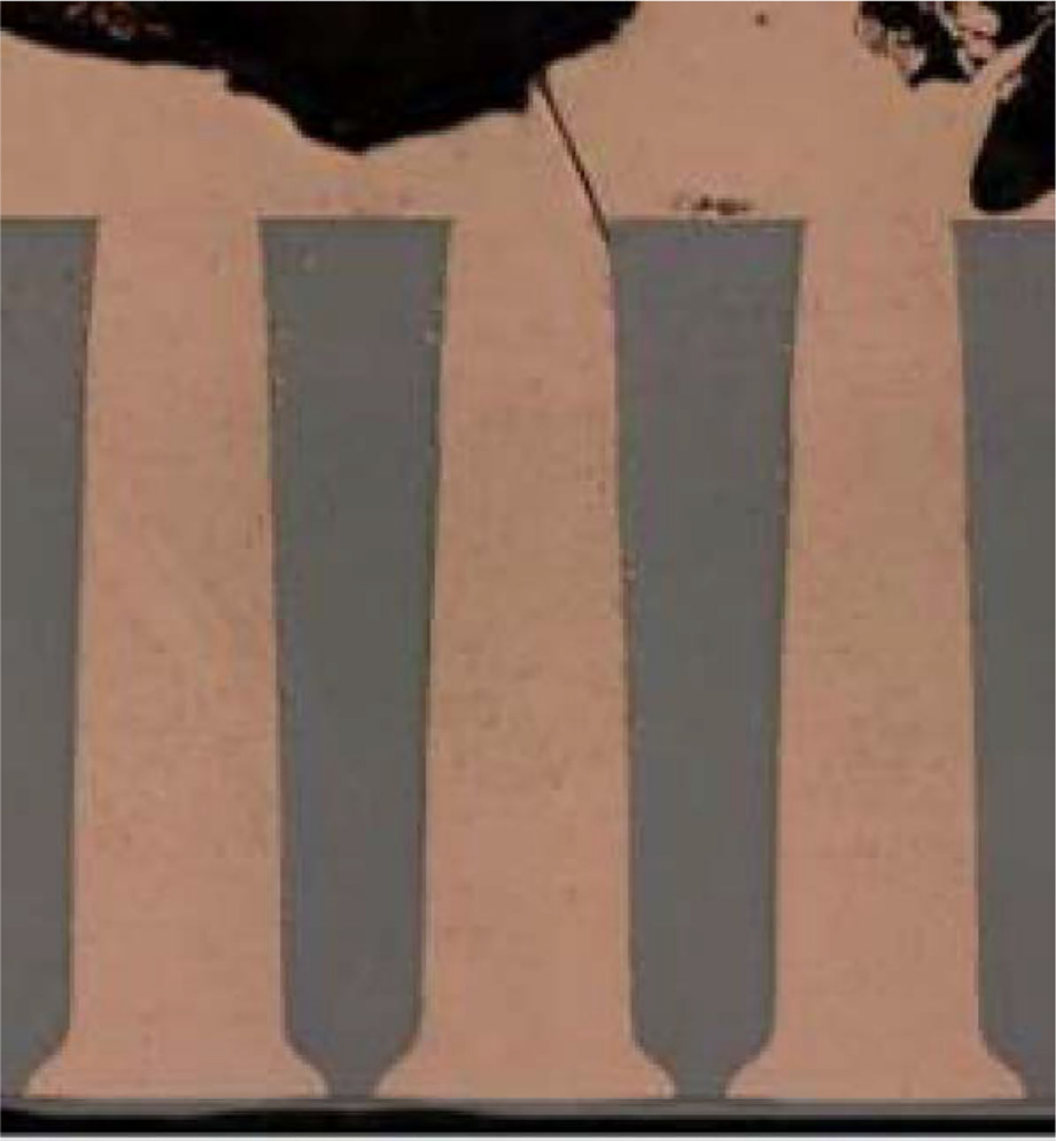
TSV filling over 8 hr at −0.62 V(SSE) in 1.25 mol/L CuSO_4_ −0.25 mol/L CH_3_SO_3_H − 1 mmol/L Cl^−^ electrolyte with 50 μmol/L poloxamine Tetronic 701. The vias were electroplated on approximately 1 cm by 2 cm samples and rotated at 400 rpm. Deposition clearly initiated from the bottom of the features although several ended with some voiding within the deposited Cu (not shown).

**Figure 9.**
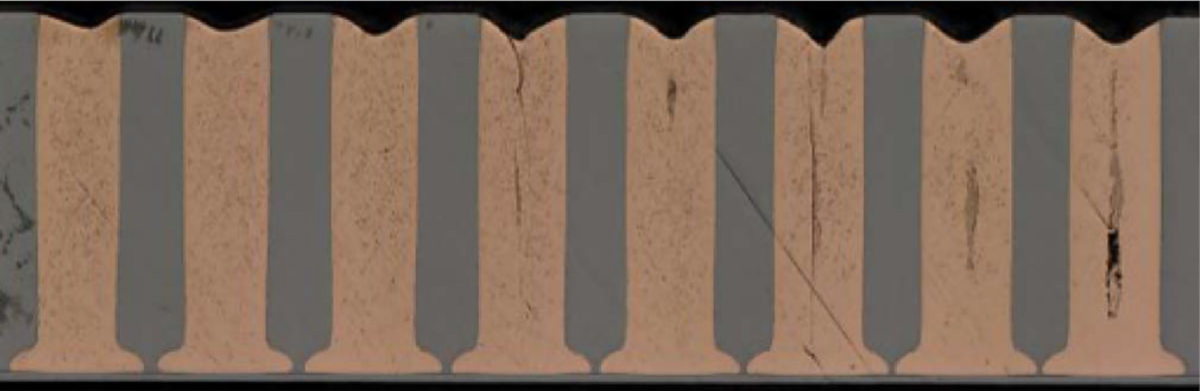
TSV filling after 20 hr in 1.25 mol/L CuSO_4_ −0.25 mol/L CH_3_SO_3_H - 1 mmol/L Cl^−^ electrolyte with 50 μmol/L poloxamine Tetronic 701. The vias were electroplated on approximately 1 cm by 2 cm samples at −0.62 V(SSE) in solution that was nominally static throughout deposition. All the TSVs contain a central seam void reflecting conformal deposition within the recessed features; seams disappear toward the left due to polishing that is not parallel to the features as is also reflected in their narrowing toward the left.

An example of complete TSV filling after 8 hrs at −0.62 V and 400 rpm in 1.25 mol/L CuSO_4_ −0.25 mol/L CH_3_SO_3_H - 1 mmol/L Cl^−^ electrolyte with 50 μmol/L poloxamine Tetronic 701 is shown in Figure 8. The influence of convection, or more precisely its absence, is captured in Figure 9, where the TSV were plated under nominally quiescent conditions. The deposition time was 20 hours, and the deposition is confined to the features. However, a central void is evident in the vias shown on the righthand side that capture the full via diameter. The miscut is also evident in the variation of the growth front across the array. Looking to the future, optimization of the polyether-halide chemistry along with the growth regulation modes should enable robust and timely filling of large scale TSVs. Galvanostatic control is also currently under examination.^21^ Significantly, while the current density may or may not be selected to vary with time, based on understanding of the mechanism^5,6^ it must be a function of the patterning, increasing with the recessed area that is to be filled.

## Summary

In this work, a fabrication facility friendly, single-additive acid sulfonic acid chemistry was successfully implemented to fill conductive large scale TSVs with void-free Cu deposited by a bottom-up process. Deposition was performed as a function of fixed applied potential in an electrolyte comprised of 1.25 mol/L CuSO_4_, 0.25 mol/L CH_3_SO_3_H, 1 mmol/L KCl and micromolar concentration of a poloxamine, Tetronic 701 (Mn ~3600). The 600 μm deep and nominally 125 μm wide TSVs were nearly fully filled within 6 hours, corresponding to a plating rate of almost 100 μm/hr or 75 mA/cm^2^. Deposition was performed with rotating rectangular wafer fragments in a manner analogous to a spinning helicopter blade. Growth front profiles and fluid dynamics calculations reveal that vortex formation toward the top of the vias impacts growth front evolution during the later stages of filling. These TSVs were filled on 2 cm by 1 cm wafer pieces while controlling the boundary layer thickness through rotation of the helicopter blade-like specimens. Work is underway to examine the efficacy of galvanostatic control for filling TSVs on a full wafer using the same polyether-halide additive chemistry.

## Figures and Tables

**Figure 1.**
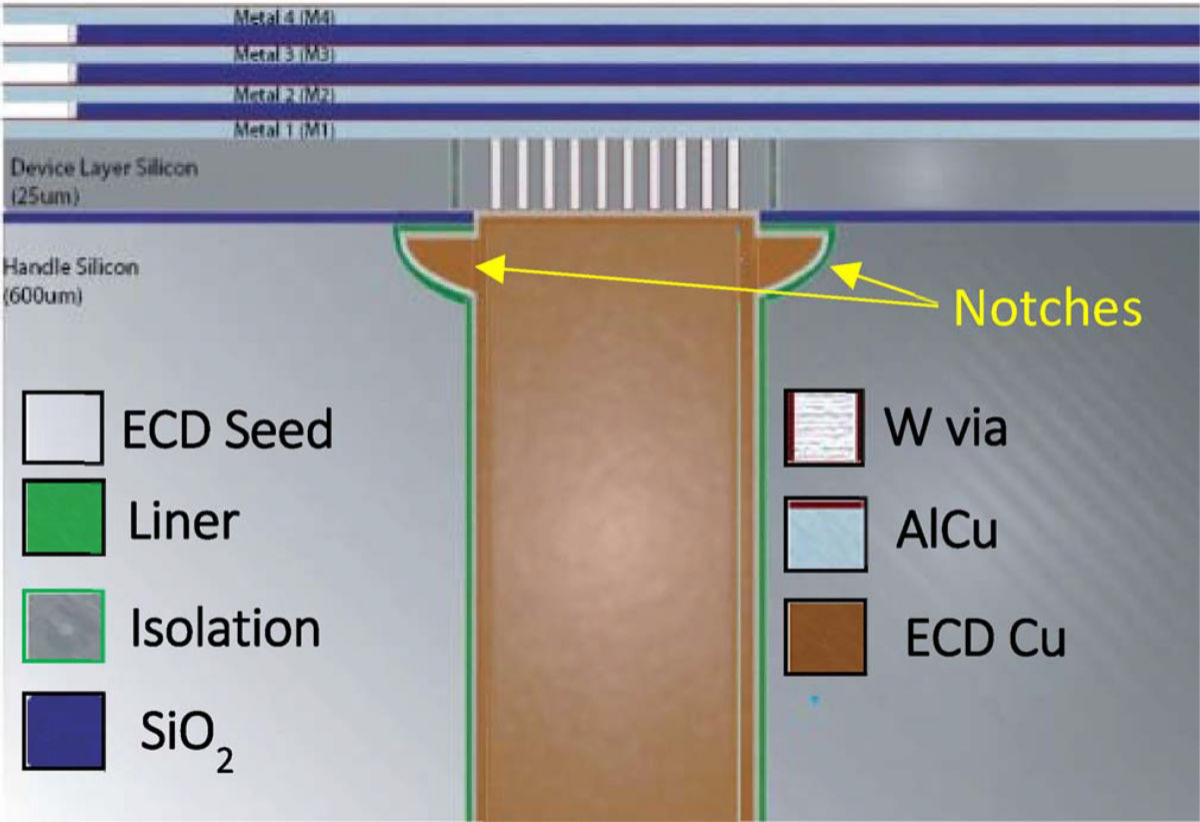
Integration architecture of the mesoscale Cu TSV, integrated with a device fabricated on a silicon-on-insulator substrate.
